# Deployment of a real-time prostate cancer confirmation system with Raman spectroscopy: fine-tuning versus test-time adaptation of 1D CNNs

**DOI:** 10.1117/1.BIOS.2.3.032706

**Published:** 2025-09-18

**Authors:** David Grajales, William T. Le, Victor Blanquez-Yeste, Frédérick Dallaire, Feryel Azzi, Guila Delouya, Dominique Trudel, Frédéric Leblond, Cynthia Ménard, Samuel Kadoury

**Affiliations:** a Polytechnique Montréal, Montréal, Québec, Canada; b Centre Hospitalier de l’Université de Montréa (CHUM), Montréal, Québec, Canada; c Institut du Cancer de Montréal, Montréal, Québec, Canada

**Keywords:** prostate cancer analysis, Raman spectroscopy, convolutional neural networks, test-time adaptation, robotic guidance

## Abstract

**Significance:**

Prostate cancer (PCa) confirmation during needle-based procedures is limited by the lack of intraoperative diagnostic tools. Raman spectroscopy (RS), combined with classification models, offers a promising solution for real-time tissue characterization, potentially improving sampling accuracy and therapy guidance. However, such models require tissue- and organ-specific data, making deployment in studies challenging due to limited data availability.

**Aim:**

The aim is to develop a one-dimensional convolutional neural network (1D-CNN) for real-time PCa detection using RS on prospectively collected *ex vivo* data, leveraging multi-organ pre-training and evaluating two domain adaptation strategies.

**Approach:**

A ResNet-based 1D-CNN was trained for binary cancer/normal tissue classification. We implemented a pre-training strategy using retrospective RS data from brain, breast, and prostate (202 patients), along with pre-trained bacterial models, followed by efficient fine-tuning and test-time adaptation (TTA) to adapt to unseen domains.

**Results:**

Prospective RS data were acquired using a robotic system from 10 PCa patients (two to five biopsies each). The fine-tuned model achieved 0.76 area under the receiver operating characteristic curve, 0.79 accuracy, 0.83 sensitivity, and 0.72 specificity, outperforming support vector machines. TTA improved predictions when labels were unavailable.

**Conclusions:**

Pre-trained 1D-CNNs combined with efficient fine-tuning or TTA enable accurate PCa detection in small-cohort settings using real-time RS.

Statement of Translation This work utilizes Raman spectroscopy and transfer learning strategies in a robot-assisted workflow to enable real-time prostate cancer detection. Fine-tuning and test-time adaptation of one-dimensional convolutional neural network models enable effective classification in small-cohort settings, supporting the clinical translation of Raman-spectroscopy-based tools for intraoperative decision-making.

## 1 Introduction

In targeted cancer therapies for prostate cancer (PCa), spatially resolved biopsy sampling with rapid online cancerous tissue confirmation stands to improve diagnosis, prognostication, and guided therapies.^1^ Although several methods have been explored for intraoperative cancer confirmation, including mass spectrometry for breast cancer margin detection,^2^ micro-ultrasound for PCa,^3^ and fast freezing techniques, these tests remain invasive, resource-intensive, time-consuming, or expertise-dependent. As a result, the quest for a reliable, non-destructive method for immediate cancer confirmation—which can be used, for instance, to guide biopsy sampling, radioactive seed implantation in brachytherapy, or surgical margin characterization during tumor resection—remains a critical objective in enhancing the precision of targeted therapies.

Raman spectroscopy (RS) is a label-free optical imaging technique that provides real-time molecular signatures based on the inelastic scattering of light. When tissue is illuminated by a laser, most photons are elastically scattered, but a small fraction undergoes energy shifts due to interactions with molecular vibrational modes (Raman effect). These energy shifts generate a spectrum that reflects the molecular composition of the tissue. This information can be exploited to detect various diseases, including Alzheimer’s, cardiovascular conditions, and cancer.^4^^,^^5^

Particularly in PCa, RS has been used to differentiate prostatic and extraprostatic tissue (with a probe integrated into a da Vinci surgical robot during radical prostatectomy)^6^ and for the detection and grading of cancer. An *in vivo* PCa detection study reported an accuracy of 0.72 using RS alone and 0.83 in a multi-modal approach (RS + multiparametric MRI-based features).^7^ On the other hand, in a large cohort *ex vivo* study with 84 patients, RS allowed differentiating cancer grades with a sensitivity of 0.90 and specificity of 0.80.^8^ However, prostate RS faces significant challenges that hinder its effectiveness in clinical applications.^4^ One major issue is the significant autofluorescence in prostate tissue compared with other organs, which can limit the quality of an already weak signal.^5^^,^^9^^,^^10^ Another challenge is the accurate assignment of site-specific ground truth labels, as opposed to core-level diagnosis, which directly affects the performance of classification models and sometimes complicates data acquisition (e.g., requiring ink marking steps).^10^^,^^11^ Finally, small cohorts constitute a significant obstacle to obtaining high-performance models, as they limit the complexity of the models that can be used and their generalizability.^3^^,^^4^ Addressing these challenges is crucial for enhancing the diagnostic potential of RS in PCa.

Across medical (*in vivo* and *ex vivo*) and non-medical applications, RS-based sample classification is commonly performed using machine learning methods such as support vector machine (SVM) or logistic regression, often combined with dimensionality reduction (e.g., principal component analysis) or feature selection (e.g., Lasso regression) due to the signal’s high dimensionality.^4^^,^^8^ Although successful applications have been reported, including Zhang et al.’s breast cancer cell line differentiation with 0.96 accuracy using SVM and principal component analysis,^12^ these approaches lack automated task-specific feature extraction, which can be time-consuming or experience-dependent.^13^^,^^14^

In the past decade, deep learning (DL)-based methods have emerged as a promising alternative for classification tasks. Although convolutional neural networks (CNNs) are traditionally applied to two-dimensional (2D) data such as images and videos for classification, detection, or segmentation, one-dimensional convolutional neural network (1D-CNN) adaptations are less resource-intensive and well-suited for silent peak detection and automated feature extraction on high-dimensional 1D signals (e.g., time series or spectra).^14^^,^^15^ These capabilities have facilitated the adoption of 1D-CNNs in various medical applications, including electroencephalography (EEG).^16^ Although their use in RS remains limited, promising results have been reported. Ho et al.^17^ achieved 0.82 accuracy in identifying 30 bacterial pathogens in patient isolates (i.e., clinical strains), even with low-quality RS signals, using pre-training and fine-tuning strategies.

Recent advancements in DL have explored the use of data from related domains to mitigate data scarcity in specialized tasks. In image analysis, models pre-trained on large-scale natural image datasets (e.g., ImageNet with 1.28M images) are frequently adapted to medical imaging tasks with far fewer samples (often fewer than 600 images).^18^ Even foundation models such as MedSAM, trained on over 1M medical images for universal segmentation, are initialized with weights from SAM, a model trained on natural images.^19^ This transfer learning approach enables models first to learn generalizable representations from abundant high-quality data and then adapt—through fine-tuning, domain adaptation, or test-time adaptation (TTA)—to specific downstream tasks.^2^^,^^16^^,^^20^^,^^21^ When ground truth labels are available, supervised fine-tuning is suitable for updating specific layers of the model to adapt to the target dataset. In contrast, when labels are unavailable, TTA allows adaptation during inference, based on an unsupervised entropy minimization for each new measurement, enabling the model to mitigate domain shifts in real time without prior exposure to the target dataset.^16^^,^^20^^–^^22^ Given the success of these strategies in imaging, we explore their potential for RS-based tissue classification by leveraging data from related domains that share the same spectroscopic technique and biological features.

In this study, we propose an approach leveraging previously acquired multi-organ cancer spectra (202 retrospective cases) for pre-training a 1D-CNN-based classification model, followed by an adaptation step to prospective prostate spectra. We compare an unsupervised (TTA) and a supervised (efficient fine-tuning) adaptation strategy, tailored to different scenarios based on ground truth availability. A robot-assisted RS framework was deployed in the procedure room to collect prospective PCa data (target dataset). Our complete approach aims to address two of the challenges of PCa detection: accurate labels and limited specific data from 10 prospective patients, which ultimately contribute to improved diagnostic capabilities and precision in targeted therapies.

## 2 Materials and Methods

### 2.1 Retrospective Datasets

To enable the use of recent pre-training methods in DL and to increase the available development datasets, three retrospective cohorts (Table 1) from in-house biobanks—containing processed RS and histopathological analysis from different anatomical sites—were combined into a single multi-organ cancer confirmation dataset. The three retrospective datasets are as follows.

**Table 1:** Characteristics of the available brain, breast, and prostate cancer patient cohorts. Figures are presented either as counts (percentage) or medians (range).

	Retrospective			Prospective
	Brain (n=67)	Breast (n=20)	Prostate (n=115)	Target prostate (n=10)
Cohort				
Sex (female)	31 (46.3%)	20 (100%)	0 (0%)	0 (0%)
Median age at diagnosis	64 (52 to 74)	67 (54 to 77)	68 (64 to 74)	66 (63 to 72)
Acquisitions				
Measurements per patient	18 (1 to 80)	6 (1 to 25)	2 (2 to 75)	10 (7 to 18)
Accumulations per spectrum	20 (20 to 20)	10 (10 to 10)	35 (20 to 50)	10 (10 to 10)
Site label distribution				
Normal	420 (42.6%)	59 (34.9%)	3066 (87.6%)	45 (43.3%)
Cancer	565 (57.4%)	110 (65.1%)	435 (12.4%)	59 (56.7%)

#### 2.1.1 Brain

An open-brain surgery dataset (glioblastoma, brain metastases, or meningioma) where samples containing >90% cancer cell burden were labeled as cancer, and as normal if no tumor cells were present; the rest were discarded,^23^ resulting in 985 measurement samples available for analysis from 67 patients.

#### 2.1.2 Breast

A breast-conserving surgery dataset where samples with >80% cancer cells were labeled as cancer and samples with >80% normal cells were labeled as healthy; the rest were discarded,^24^ resulting in 169 measurement samples available for analysis from 20 patients.

#### 2.1.3 Prostate

An *in vivo* dataset acquired during high-dose-rate brachytherapy (19 patients) and an *ex vivo* dataset on radical prostatectomy (96 patients). Cancer labels were assigned to samples with a grade group according to the International Society of Urological Pathology (ISUP GG) ≥1.^7^ Although these data come from the same organ as our prospective target dataset, the instruments, protocols, and sample characteristics (e.g., specimen thickness and inspected depth) are different.

For these retrospective datasets, methods were employed to reduce alignment uncertainty between the inspected site and the ground truth, such as averaging cell counts across different regions of the specimen, using grids and ink for aligning macroscopic images with hematoxylin and eosin staining (H&E) images or employing an electromagnetic/ultrasound navigation system.^7^^,^^10^^,^^23^^,^^24^ Overall, 4655 RS measurements were available for analysis from 202 patients. The brain and breast datasets were included alongside prostate because they were available in our biobanks, acquired using similar RS systems, and offered higher signal quality with lower autofluorescence than prostate tissue. They also contained a higher proportion of tumor spectra.

### 2.2 Prospective Dataset

**Fig. 1:**
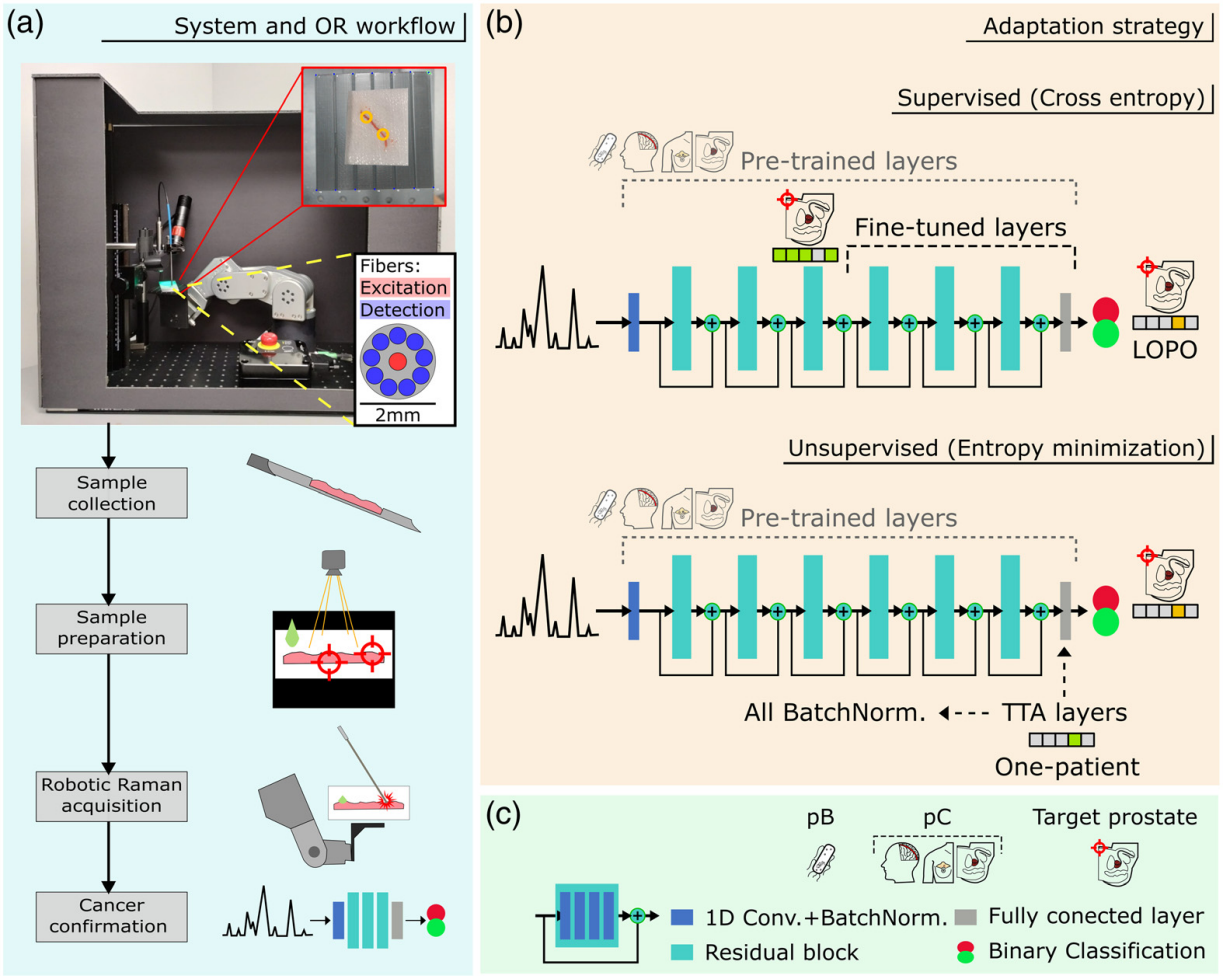
System and model overview. (a) Robot-assisted Raman acquisition system, including the robotic arm, camera, and optical probe; insets show a front-view schematic of the probe tip (yellow), a close-up of the tissue positioning platform (red), and the intraoperative workflow. (b) Schematic illustration of the 1D-CNN (ResNet-based) model used for PCa detection, showing the two adaptation strategies; LOPO: leave-one-patient-out. (c) Symbol legend: pB, bacteria pre-training; pC, cancer pre-training (retrospective data from brain, breast, and prostate).

The target prostate dataset was acquired prospectively in 10 patients with histologically confirmed PCa diagnosis (stage I, organ-confined disease with no lymph node or distant metastases) who underwent high-dose-rate brachytherapy. They were enrolled in a prospective clinical trial approved by the Research Ethics Board and recruited between April and December 2024. The system used for data acquisition in the procedure room consists of a robotic arm and an optical component [Fig. 1(a)]. The optical measurements were performed on two to five biopsy cores per patient following the workflow evaluated in previous stages of this project^14^ and summarized in Sec. 2.3.

### 2.3 Instruments and Workflow

Prior to every intervention in the procedure room, the robot + camera subsystem was calibrated with fiducial markers to ensure targeting accuracy during sample handling. The manually excised tissue (biopsy core) was placed on the platform attached to the robot, and histopathology-compatible ink marks were used to determine the specimen’s orientation (for ground truth purposes). Once in the camera’s field of view, an image of the biopsy sample is displayed, enabling the selection of RS measurement sites. The semi-automated pipeline started when the robot moved the platform to place each sample’s site under the fixed RS probe. Finally, as RS is a non-destructive technique, the tissue was subsequently fixed in formalin for standard histopathological procedures. After processing, each spectrum consisted of 1174 Raman shift feature points, presenting a high-dimensional data vector with a sample size of 104 spectra.

#### 2.3.1 Robotic system and calibration

A 6-degree-of-freedom (DOF) robotic arm (Mecademic, Montreal, Canada), which integrates a built-in controller and a Python API enabling high precision and accuracy in motion control, was used for biopsy core handling. Images acquired with a Logitech-C920 camera were used to select the sample’s sites to be characterized and to find the correspondence between the pixel coordinates in the image (m,n) and the coordinates on the robot platform (x,y), based on known fiducial, using a conic 2D–2D mapping strategy. Details on mapping strategy selection and robot control can be found in Ref. 14. By leveraging the robot’s precise positioning, the distance between each characterized site and a reference ink mark is accurately determined, enabling the assignment of site-specific ground truth to Raman spectra. This approach reduces the dependency on core-level diagnoses and mitigates potential mislabeling caused by tissue heterogeneity.^11^

#### 2.3.2 Raman optical system and measurements

A near-infrared RS instrument (Sentry 1000-R, Reveal Surgical Inc., Montreal, Canada) was used to obtain spectral fingerprints from the samples. The complete system consisted of a hand-held Raman probe (EmVision, Loxahatchee, Florida, United States), a 785-nm light source (Innovative Photonic Solutions, Plainsboro Township, New Jersey, United States), a spectrometer (Newton model, Andor Technology, Belfast, Ireland), and a control laptop with custom software packages ORAS and ORPL.^25^ The 12-cm probe contains a central excitation fiber surrounded by nine collection fibers of 500  μm core diameter and optical filters in the probe tip to minimize signals from fiber materials and tissue autofluorescence and to ensure contact to interrogate 0.5 mm diameter spots.^23^ All raw measurements were acquired, in contact, inside a protection box, covering a range from 400 to $2000\;{\mathrm{cm}}^{-1}$, with auto-exposure control set to a range from 100 to 1000 ms for 10 accumulations; the detector was pre-cooled at −70°C. The laser power was limited to 100 mW; all exposures were kept under twice the maximum permissible exposure for skin, as defined by ANSI Z136.3 standards, and histopathologists confirmed the absence of thermal damage in all samples.^26^ The applied preprocessing consists of averaging the accumulations, background and cosmic rays subtraction, instrument response correction (NIST Raman standard—SRM2214), autofluorescence removal (BubbleFill^25^), normalization, and x-axis calibration (Raman shift).

### 2.4 Proposed RS Classification Models

Given the recently demonstrated importance of pre-training DL models when extracting relevant features on target data, we leverage the publicly available 1D-CNN weights from the works of Ho et al.^17^ for bacterial pathogens identification, trained on ∼60,000 RS samples. As their approach used a similar RS acquisition protocol as in this study, we hypothesized that the early feature extraction portion of the model trained on biological samples would directly benefit the adapted PCa confirmation task.

This study used a ResNet-based model for PCa confirmation, capitalizing on the improved gradient propagation and training stability attributed to residual blocks.^17^^,^^20^ The model, built following the architecture used by Ho et al.,^17^ consists of an initial 1D convolutional layer (64 convolutional filters), six residual blocks, and a fully connected layer at the end for binary classification (binary cross-entropy loss). Residual blocks have four 1D convolutional layers and a residual connection (shortcut) between the input and output of the block [Figs. 1(b) and 1(c)].

The search for hyperparameters such as epochs and learning rate was conducted using a one training–validation (60% to 20%) split optimized for accuracy. The CNN underwent training for 25 epochs (selected after searching 5 values in the range 25 to 100), with early stopping after 8 consecutive epochs without improvement in validation accuracy; the Adam optimizer was used with a learning rate set at 0.001 (selected after searching four values in the range 0.0001 to 0.01). Each convolutional layer utilized a kernel size of 5 and a stride of 2 for downsampling purposes. The models (1.25M parameters) were implemented in PyTorch and trained on an NVIDIA GPU with 15 GB of RAM.

A two-stage pre-training framework was used to address the challenges of adapting DL models to limited data: (i) the abovementioned weights initialization [bacteria pre-training (pB)] and (ii) pre-training on the combined retrospective heterogeneous cancer dataset from diverse RS systems, organs, and tissue size [cancer pre-training (pC)]. The pre-trained model was adapted to the target prostate dataset under two different scenarios.

Supervised adaptation—spectra and ground truth available for fine-tuning the specific classification task: Building on Zhang et al.’s^16^ adaptive transfer learning strategies,^16^ our approach selectively fine-tuned the pre-trained model. Specifically, the first half of the model remained unchanged, whereas the last three residual blocks and the fully connected layer were adapted to the target data, as shown in Fig. 1(b), using a binary cross-entropy loss. This approach of optimizing transfer learning reduces trainable parameters by half and naturally mitigates overfitting without relying on dropout layers.^18^

Unsupervised adaptation (TTA)—spectra available at the moment of acquisitions, with no ground truth: Here, moving toward unsupervised learning, TTA was applied based on entropy minimization of the model’s prediction ($\hat{y}$) of every new measurement with the objective function H(y^)=−∑cp(y^c)log p(y^c), for the probability ${\hat{y}}_{c}$ of class c.^21^ In this process, specific layers of the model (final fully connected layer and BatchNorms) were adapted in a limited number of steps (5), looking to reduce the unsupervised loss, then produce a classification prediction.^21^^,^^27^

### 2.5 Comparative Methods

#### 2.5.1 Baseline SVM

A baseline SVM was implemented for comparative purposes. Feature selection is an important part of using SVMs with a limited number of high-dimensional samples, such as RS. For the prostate target dataset, a three-step method (amount of variation >0.03; correlation with the target >0.9; Lasso regression for a maximum of 10 features) was applied to the training set of each fold, considering every data point in the spectra as a feature.^7^

Previous works have shown the potential of specific Raman peaks for PCa detection. Peaks associated with phenylalanine ($994\;{\mathrm{cm}}^{-1}$ and $1007\;{\mathrm{cm}}^{-1}$); with collagen, DNA, or RNA ($1334\;{\mathrm{cm}}^{-1}$); and with DNA/RNA, proteins, and phospholipids ($1766\;{\mathrm{cm}}^{-1}$, and $1772\;{\mathrm{cm}}^{-1}$) were selected in an *in vivo* study.^7^ In an *ex vivo* study, the peaks at $881\;{\mathrm{cm}}^{-1}$ (tryptophan), 1307 to $1310\;{\mathrm{cm}}^{-1}$ (collagen), $1396\;{\mathrm{cm}}^{-1}$ (β-carotene), $1583\;{\mathrm{cm}}^{-1}$ (phenylalanine), and $1602\;{\mathrm{cm}}^{-1}$ (phenylalanine, tyrosine, and tryptophan) were identified as biomarkers.^8^ These two groups of selected features were also used for the comparative SVM model.

#### 2.5.2 Random forest

A random forest (RF) classifier was also implemented and trained using scikit-learn with 100 trees (n_estimators = 100), maximum depth of 5 (max_depth = 5), considering all features at each split and no correction for class imbalance.

### 2.6 Experimental Methodology and Evaluation Metrics

#### 2.6.1 Pre-training strategy selection

For the pB, the clinical pre-trained model from Ref. 17 was used for initializing all network layers. pC involved all retrospective datasets (i.e., excluding prostate target dataset) divided into 60%−20%−20% for training–validation–testing. The classification performance of both strategies, and their combination (pB+pC), was tested using the target prostate data (prospective dataset), with and without fine-tuning.

#### 2.6.2 Comparative analysis of adaptation strategies

The proposed adaptation strategies (supervised and unsupervised) were tested and compared with SVM and RF. For fine-tuning adaptation (F), a 10-fold leave-one-patient-out (LOPO) cross-validation was performed on the prostate target dataset, where nine patients’ data were used for training in each fold, whereas the remaining patient was reserved for validation once. Equivalently, in TTA, one patient at a time was used to adapt the pre-trained model (the adaptation of the model based on one patient does not affect the other patients’ results).

#### 2.6.3 Evaluation and metrics

A leave-one-patient-out scheme was used to train the SVM and RF models and to adapt the 1D-CNN models via fine-tuning. Performance was assessed using the area under the receiver operating characteristic (ROC) curve (AUC), accuracy, sensitivity, and specificity. To ensure statistical reliability, all experiments were repeated five times, with results reported as mean and standard deviation [mean (SD)]. Statistical significance was evaluated using Student’s t-test (p<0.05), and confidence intervals (CIs) were included to quantify result variability.

## 3 Results

### 3.1 Data Acquisition

**Fig. 2:**
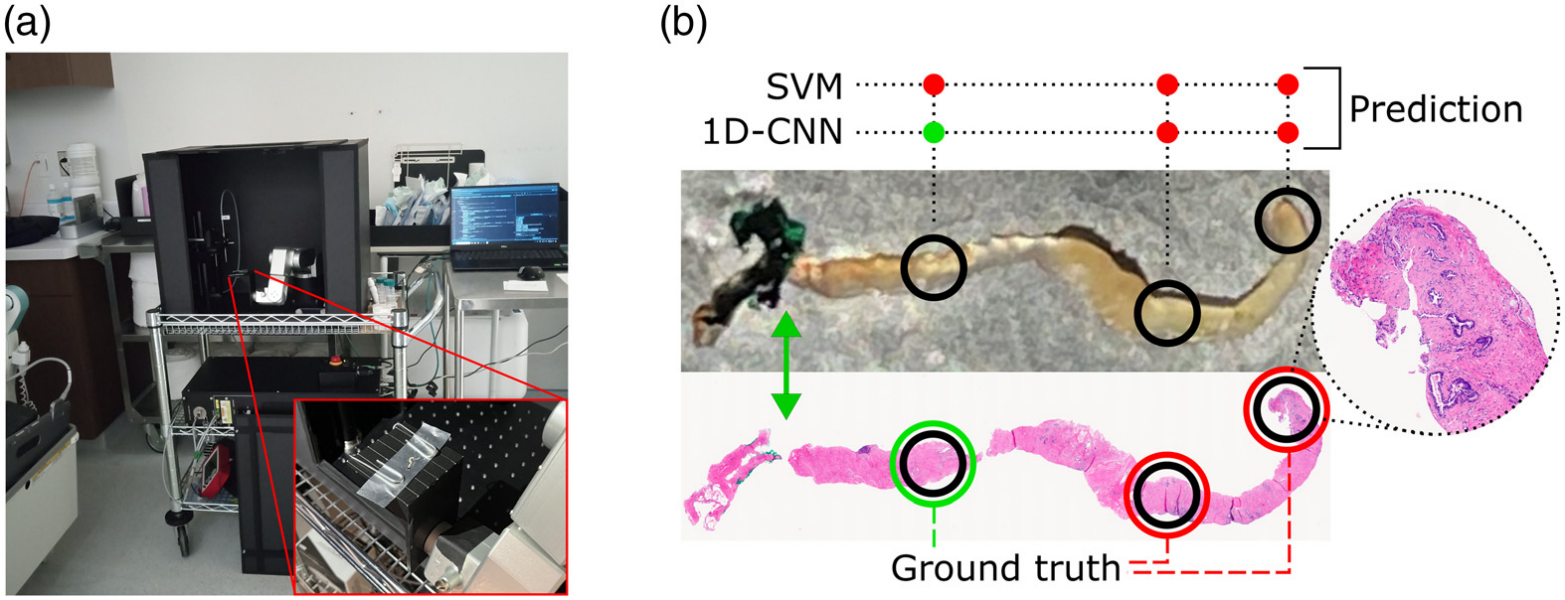
System deployed in the procedure room for real-time tissue characterization and classification. (a) Robot-assisted RS system (protection box, robotic arm, and optical components) in the procedure room, with a close-up of a biopsy sample on the platform. (b) Sample prediction results of real-time biopsy tissue classification. Photograph and corresponding histopathological slide of a biopsy core. Black circumferences: characterized sites; colored circumferences: diagnosis in the pathology report (red: cancer); colored circle: prediction of each model (red: cancer); green arrow: beginning of the ink mark.

The system was deployed in the procedure room and collected data from 10 PCa patients (Table 1), with two to five biopsies obtained per patient. The entire process to characterize one biopsy core (including three to six RS measurements per sample) took between 4 and 5 min, excluding biopsy acquisition time. The probe was positioned in direct contact with the tissue to optimize the signal-to-baseline ratio while minimizing interference from the support material. The DL models were deployed to assess inference timing. The time between tissue collection and the first prediction was <3  min, including sample preparation, spectral acquisition, processing, and inference—performed in 584 ms on average. The time between biopsy and formalin fixation was <12  min for all samples. The histopathology results of each core, obtained subsequently, were used as ground truth (cancer labels were assigned to sites with ISUP GG ≥1). Images and sample predictions are presented in Fig. 2.

**Fig. 3:**
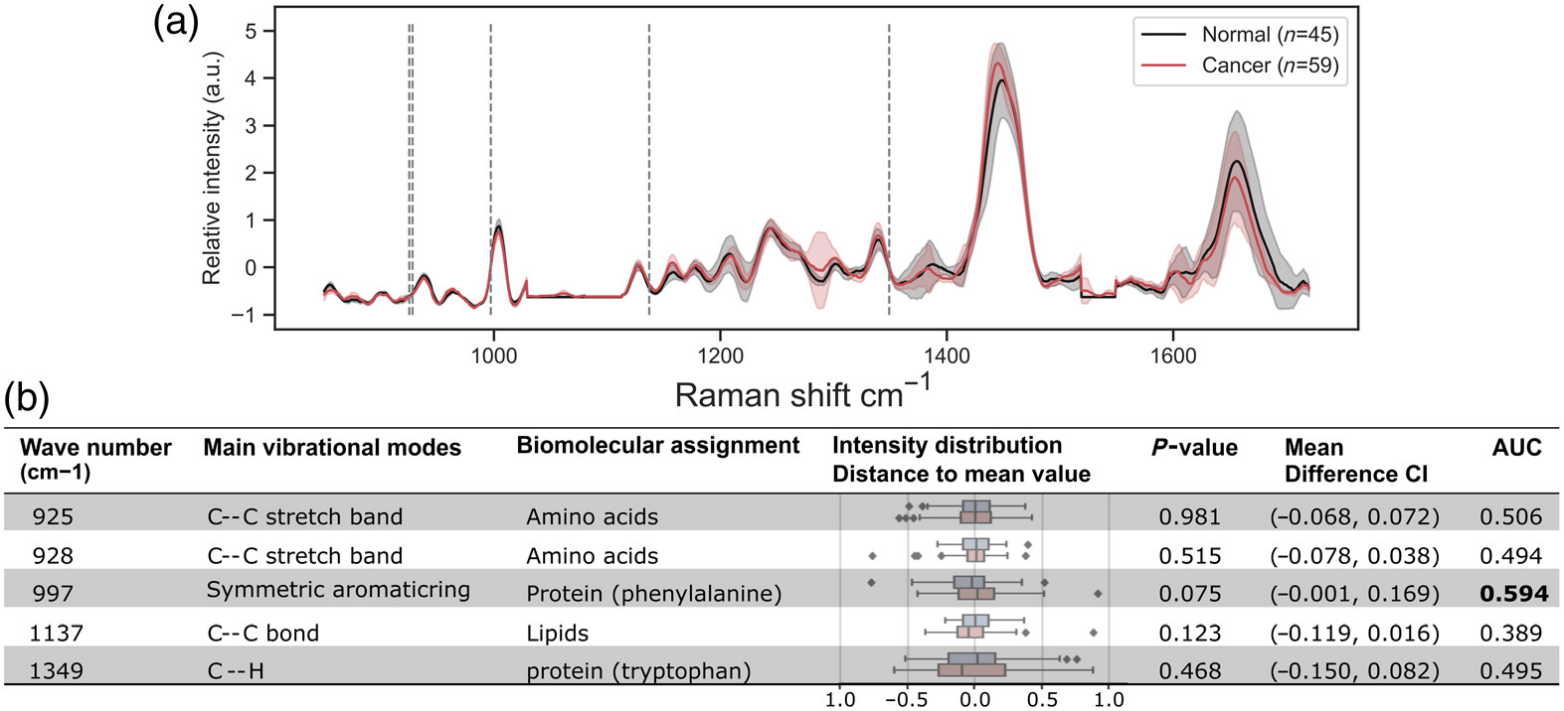
(a) Average Raman spectra with their variance from the prospective target prostate *ex vivo* dataset (10 patients). The vertical dotted lines indicate the features selected for training the SVM models. (b) Peak locations and corresponding biomolecular assignments are listed. Statistical and classification metrics, including intensity distribution distance, p-value (cancer versus normal), mean difference with CI, and AUC, to assess the discriminatory potential of individual Raman shifts for prostate cancer detection.

Figure 3(a) shows the post-processed Raman spectra data for the 10 prospective cases (target prostate dataset) and the features that were selected for SVM training. In an univariate analysis, statistical values and classification metrics were calculated to evaluate the discriminatory potential of each peak separately. Based on the p-value and mean difference CI, both classes mostly overlap (i.e., all p>0.05 and all CIs include 0). In terms of classification, the best AUC was obtained with the intensities of the phenylalanine-associated peak ($997\;{\mathrm{cm}}^{-1}$).

### 3.2 Pre-Training Strategy Selection

**Fig. 4:**
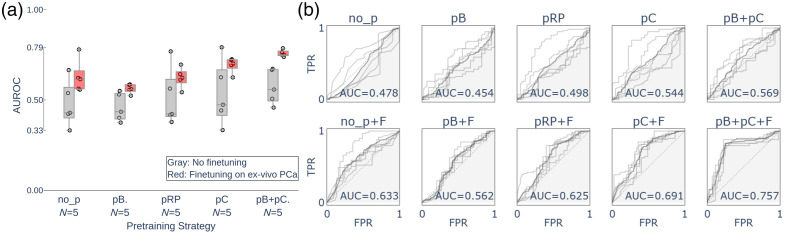
Comparison of pre-training strategies for the proposed 1D-CNN model for PCa *ex vivo* confirmation using the target dataset (10 patients, leave-one-patient-out, with 5 repetitions). (a) Boxes show the interquartile range (25% to 75%), whereas lines show the minimum and maximum. (b) ROC curve for all model variants. F, fine-tuning; no_p, no pre-training; pB, bacteria pre-training; pC, cancer pre-training (retrospective brain, breast, and prostate); pRP, pre-training using only retrospective prostate data.

The following experiment assessed the impact of pre-training strategies (bacterial spectra initialization and cross-organ transfer) with or without fine-tuning, compared with the base model without pre-training (no_p). Results are shown in Fig. 4. Overall, fine-tuning not only increased the mean AUC but also reduced the high inter-run variability.

The model combining both pre-training strategies and fine-tuning (pB+pC+F) and the non-initialized version (pC+F) exhibited superior overall performance. The difference in AUC among them was significant (pC+F − pB+pC+F =−0.06 AUC, p=0.012, CI=[−0.111,−0.037]); further analysis showed that pB+pC+F achieved better accuracy [0.79 (0.03)] and specificity [0.72 (0.04)] than pC+F [0.69(0.02), 0.51(0.06)] with both differences being statistically significant (p<0.001 and p<0.001). No significant difference in sensitivity was observed among the models (pC+F − pB+pC+F =−0.01, CI=[−0.041,0.027]).

Another key element of the initialization (pB) is the reduction in pre-training execution time. When calculating the mean number of epochs before the early stop, a value of 14 (range: 9 to 17) was found for pB+pC+F, versus 18 (range: 15 to 23) for pC+F, representing a 26% reduction in execution time during pre-training, approximately −54  min per test (five repetitions).

An additional test using only prostate retrospective data for pre-training (pRP+F) resulted in lower average performance and higher variability compared with the pC+F model (pRP+F − pC+F =−0.07 AUC, CI=[−0.122,−0.012,]), which was pre-trained on the same data plus brain and breast data. Despite the closer domain similarity, pRP+F showed −0.13 AUC (p<0.001), −0.15 accuracy (p<0.002), −0.07 sensitivity (p<0.256), and −0.25 sensitivity (p≪0.001), compared with pB+pC+F, the approach that includes greater diversity of source data.

Based on these findings, subsequent downstream tests adopted the combined strategy of Bacteria pre-training followed by cancer pre-training (p).

### 3.3 Quantitative Analysis of Adaptation Strategies

Following the selection of the combined pre-training strategy (p), which yielded a residual misclassification rate of 28% during internal validation, the two adaptation strategies were finally evaluated on the prospective prostate dataset (10 patients). Table 2 presents the classification results across all metrics, along with those of the comparative models. The SVM results were obtained using fold-specific selected features (Fig. 3) and literature-derived feature sets,^7^^,^^8^ and the averaged values enable comparison with 1D-CNN performance.

**Table 2:** Comparative results of 10-fold cross-validation for discriminating cancer from normal tissue.

Model	Variation	AUC	Accuracy	Sensitivity	Specificity
SVM	Feat. Sel.	0.57	0.60	0.88	0.26
	Feat. from Ref. 7	0.56	0.60	0.66	0.53
	Feat. from Ref. 8	0.60	0.62	0.75	0.45
	Average	0.58 (0.02)	0.61 (0.01)	0.76 (0.09)	0.41 (0.11)
RF	—	0.61 (0.03)	0.57 (0.03)	0.63 (0.03)^*^	0.51 (0.07)
1D-CNN	p+TTA	0.60 (0.01)	0.63 (0.01)	0.67 (0.04)	0.55 (0.04)^*^
	p+F	**0.76 (0.01)** ^*^	**0.79 (0.03)** ^*^	**0.83 (0.03)**	**0.72 (0.04)** ^*^
	p+F+TTA	0.74 (0.01)^*^	0.72 (0.03)^*^	0.73 (0.06)	0.70 (0.04)^*^

* Statistically significant difference against SVM baseline (p<0.05). All figures are presented as mean (SD) of five repetitions. F, fine-tuning; p, bacteria pre-training + cancer pre-training.

The model fine-tuned after pre-training (1D-CNN+p+F) achieved +0.18 AUC, compared with the SVM baseline, with p≪0.001 and a 95% confidence interval (CI=[0.154,0.200]) excluding the origin, suggesting a statistically significant performance improvement. Similarly, it showed a significant improvement of +0.18 (p≪0.001) in accuracy and +0.31 (p=0.003) in specificity. Despite a +0.07 difference in sensitivity in favor of 1D-CNN+p+F, this was not significant (p=0.204).

The slight improvements in AUC and accuracy of the pre-trained model adapted via TTA (1D-CNN+p+TTA) over SVM (+0.02, p=0.060, p=0.081) were not statistically significant, nor was the −0.09 difference in sensitivity (p=0.113). However, the improvement in specificity was significant (+0.14, p=0.040). This highlights the potential of TTA as an unsupervised adaptation strategy, achieving performance comparable to a supervised baseline trained with labeled data from nine patients.

The AUC of the RF model was slightly better than SVM, but the difference was not significant (+0.04 AUC, p>0.124); sensitivity was the only metric where the difference between SVM and RF was significant (+0.13 in favor of SVM, p<0.040).

**Fig. 5:**
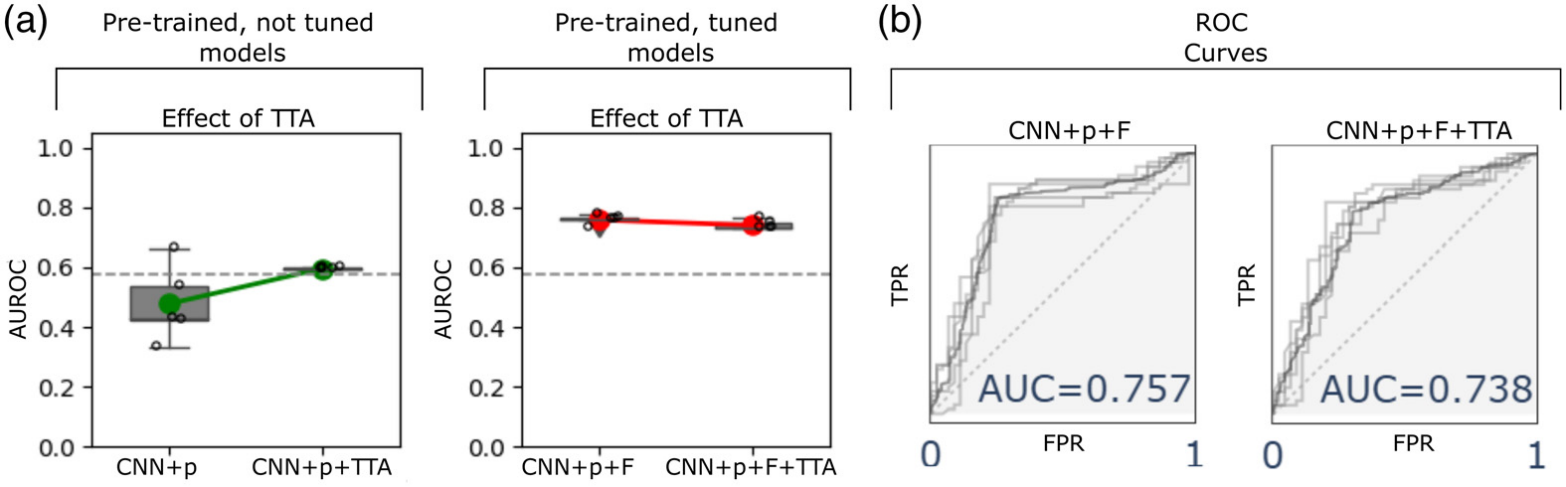
Comparison of AUC plots for the proposed models. (a) Pairwise comparison on adaptation, five repetitions for all models. Boxes show the interquartile range (25% to 75%), whereas box-whiskers show minimum and maximum values within 1.5× the interquartile range. The horizontal dotted lines indicate the mean AUC value for the SVM baseline model. (b) ROC curve for the proposed models. F, fine-tuning; p, bacteria pre-training + cancer pre-training.

To better illustrate the effect of supervised (fine-tuning) and unsupervised (TTA) adaptation, pairwise comparisons are presented in Fig. 5(a). When using a pre-trained—without fine-tuning—model (1D-CNN+p) with a considerable inter-run variability, applying TTA (1D-CNN+p+TTA) increased AUC by +0.12 (p=0.070), accuracy by +0.13 (p=0.017), sensitivity by +0.13 (p=0.192, CI=[0.007,0.329]), and specificity by +0.11 (p=0.325). In contrast, a previously fine-tuned model (1D-CNN+p+F) decreased the performance after unsupervised adaptation (1D-CNN+p+F+TTA) by −0.02 (p=0.102) in AUC, −0.07 (p=0.004) in accuracy, +0.10 (p=0.008) in sensitivity, and +0.02 (p=0.579) in specificity.

## 4 Discussion

This study explored pre-training and model adaptation methodologies for RS-based PCa confirmation in the procedure room, with prospective data from biopsy samples acquired using a novel robot-assisted workflow allowing site-specific ground truth assignment. The findings highlight the potential of leveraging multi-organ RS datasets to enhance real-time diagnostic accuracy in limited-cohort settings.

The significant variations observed in the baseline SVM results underscore the crucial impact of feature selection on model performance, revealing a strong dependence on both organ-specific and dataset-specific characteristics. The $997\text{-}{\mathrm{cm}}^{-1}$ peak, identified via the three-step selection, is notable for its presence across biological tissues (bacteria, brain, and prostate)^17^^,^^23^^,^^28^^,^^29^ and for achieving an AUC comparable to SVM, despite a non-significant class intensity difference (p=0.075).

The limited number of samples—especially healthy tissue—compromises specificity and underscores the need for robust sampling. The 0.61 accuracy of SVM models is lower than the accuracy reported in prior studies [0.72 (Ref. 7) and 0.82 (Ref. 8)], likely due to this study’s smaller cohort (n=10 versus n=19 and n=84). These results highlight the need for accurate diagnostic models using limited datasets, with automatic feature extraction.

Initializing the model with bacterial spectra (pB) reduced convergence time and inter-run variability. Multi-organ pre-training (pC) better prepared the model for the molecular signatures present in the target dataset, giving access to >200 patients and >3000 healthy spectra. Pre-training on a retrospective PCa dataset provided limited improvement. Although labels were assigned using the same ISUP-based criteria, this dataset was highly imbalanced (tumor: 435 spectra, 12.4%; normal: 3066 spectra, 87.6%) and acquired under different conditions; together with the lower number and quality of cancer spectra, these factors likely limited the benefit of prostate-only pre-training. This suggests that, despite domain similarity, the higher diversity, greater number of cancer labels, and higher quality of the multi-organ dataset (pC and pB+pC) yielded more robust and generalizable spectral representations. High-quality brain and breast spectra, with lower autofluorescence and superior cancer detection rates,^23^^,^^24^ further improved robustness. Training on diverse datasets also helps prevent overfitting.^18^^,^^22^

Once the source model was pre-trained (p), adaptation to prospective PCa data can follow two distinct strategies. If data collection is ongoing and ground truth labels are unavailable, unsupervised adaptation via TTA is the preferred option. Alternatively, if a small labeled dataset is available, fine-tuning becomes feasible. In both cases, without adequate pre-training data, adaptation would not be possible.

The proposed model for the first scenario (CNN+p+TTA) achieves comparable accuracy and sensitivity while outperforming the baseline SVM in specificity (0.55 (0.04) versus 0.41 (0.11), CI=[0.022,0.286]). Notably, the SVM required training on data from nine patients, whereas CNN+p+TTA adapted without prior exposure to the target dataset and without manual feature selection. This unsupervised adaptation is computationally efficient, enabling real-time implementation during data collection, with five fitting steps and final prediction completed in <5  s. Although TTA has been successfully applied to imaging^21^ and EEG signals,^20^ to our knowledge, its application in RS for cancer detection is novel.

For cases where labeled data are available, the fine-tuned model (CNN+p+F) demonstrates a significant improvement in AUC [0.76 (0.01) versus 0.58 (0.02) p≪0.001], accuracy (p≪0.001), and specificity (p=0.003). It also reduces inter-run variability (lower SD in all metrics except accuracy compared with the SVM). Specificity was particularly problematic for models without fine-tuning, with SVM exhibiting good sensitivity at the cost of specificity. These results yield a positive predictive value of 0.76 (0.03), which in intraoperative settings could support decision-making and personalized treatment adjustments.

Our fine-tuning approach was based on the hypothesis that the initial 1D-CNN layers act as robust feature extractors, initialized from bacterial (∼60,000 samples) and cancer (∼4600 samples) RS datasets. By freezing these lower layers and adapting only the later layers to the limited target prostate dataset, this strategy preserves the general spectral features, mitigates overfitting, and reduces computational time (−54  min per five repetitions)^16^ while effectively adapting to the PCa detection task.

In computer vision, pre-training on different domains is primarily used for feature extraction rather than classification.^21^^,^^22^ Similarly, in this study, despite differences in biological origin, bacterial and cancerous tissue spectra share fundamental Raman characteristics. This enables the CNN to learn relevant spectral representations during pre-training, which can be effectively refined for PCa detection.

The CNN+p+F model achieved a sensitivity of 0.83, representing a 13% improvement over the false-negative rates of MRI-guided biopsies.^8^ Notably, despite being implemented with only 10 patients, its performance is comparable to or better than SVM-based studies using eight times larger cohorts (e.g., 0.72 sensitivity with SVM in Ref. 8). Furthermore, the model’s sensitivity and specificity align with those reported in other organ studies, such as breast cancer, where CNN models have achieved a sensitivity of 0.86 and specificity of 0.75, despite the use of significantly larger and higher-quality datasets.^29^

The TTA approach improved performance when applied to a pre-trained—but not fine-tuned—model (5), helping mitigate distribution shifts. However, after fine-tuning, TTA reduced the overall performance. As fine-tuning had already aligned the model to the target PCa data using a supervised objective (binary cross-entropy), applying TTA modified the feature space without supervision (entropy minimization), potentially causing the model to discard relevant learned features.^27^^,^^30^ When labels were available, supervised adaptation such as fine-tuning is superior;^31^ TTA is most useful only when ground truth is unavailable; otherwise, it may be unnecessary or even detrimental.

Limitations in this study relate to the small cohort size and controlled acquisition conditions. Although using 10 patients allowed for the evaluation of adaptation strategies, the dataset may still under-represent interpatient PCa variability. All spectra were acquired using a single RS system at a single center, limiting the generalizability of the results. Although RS signals can vary across platforms due to the differences in optical design and sampling geometry, our preprocessing pipeline mitigates many of these variations by producing well-defined, interpretable spectral peaks. Combined with the adaptation strategies applied here and pre-training on multiple biobanks, this makes the approach, in principle, transferable to other systems with appropriate calibration/adaptation.^32^

Although androgen receptor status and genomic mutation data (tumor subtypes) were not collected for this cohort, both are important biological factors that can influence tumor progression and alter tissue biochemical composition, potentially affecting Raman spectral signatures.^33^^,^^34^ The absence of this information limits our ability to evaluate its specific contribution to classification performance. Future work will include multi-center data collection and system-level variability assessment, exploration of narrower spectral subranges for CNN-based classification similar to the feature selection approaches already applied to SVM models, as well as integration of real-time classification into the acquisition workflow to improve signal quality and intraoperative efficiency.

## 5 Conclusion

This study demonstrated the potential of pre-trained 1D-CNN models, leveraging multi-organ cancer spectra, to enhance RS-based PCa detection accuracy despite the limited target domain sample size. When ground truth is unavailable, TTA provides results comparable to supervised SVMs; however, when ground truth is available, supervised fine-tuning improves cancer confirmation. By integrating robot-assisted RS and efficient fine-tuning, our approach addresses critical challenges in PCa diagnosis, including accurate labeling and limited specific data. The proposed robotic-assisted workflow streamlines tissue analysis and supports the clinical translation of RS-based diagnostic tools for intraoperative decision-making.

## Data Availability

The data supporting the findings of this study are available at https://github.com/DGdaorgralo/raman-1dcnn-pca-confirmation.git. Additional information may be obtained from the authors upon reasonable request and subject to institutional ethics approval.
